# Search Methods Used to Locate Missing Cats and Locations Where Missing Cats Are Found

**DOI:** 10.3390/ani8010005

**Published:** 2018-01-02

**Authors:** Liyan Huang, Marcia Coradini, Jacquie Rand, John Morton, Kat Albrecht, Brigid Wasson, Danielle Robertson

**Affiliations:** 1 Gatton Campus, The University of Queensland, Queensland 4343, Australia; rien@live.com.sg (L.H.); j.rand@uq.edu.au (J.R.); 2 Australian Pet Welfare Foundation, Kenmore, Queensland 4069, Australia; jacquie@petwelfare.org.au; 3 Jemora Pty Ltd., Geelong, Victoria 3220, Australia, john.morton@optusnet.com.au; 4 Missing Pet Partnership, Cloverdale, CA 6105, USA; info@katalbrecht.com (K.A.); bwasson@missingpetpartnership.org (B.W.); danielle@lostpetresearch.com (D.R.)

**Keywords:** cat, pet, missing, lost, search, SNR, questionnaire

## Abstract

**Simple Summary:**

A least 15% of cat owners lose their pet in a five-year period and some are never found. This paper reports on data gathered from an online questionnaire that asked questions regarding search methods used to locate missing cats and locations where missing cats were found. The most important finding from this retrospective case series was that approximately one third of cats were recovered within 7 days. Secondly, a physical search increased the chances of finding cats alive and 75% of cats were found within a 500 m radius of their point of escape. Thirdly, those cats that were indoor-outdoor and allowed outside unsupervised traveled longer distances compared with indoor cats that were never allowed outside. Lastly, cats considered to be highly curious in nature were more likely to be found inside someone else’s house compared to other personality types. These findings suggest that a physical search within the first week of a cat going missing could be a useful strategy. In light of these findings, further research into this field may show whether programs such as shelter, neuter and return would improve the chances of owners searching and finding their missing cats as well as decreasing euthanasia rates in shelters.

**Abstract:**

Missing pet cats are often not found by their owners, with many being euthanized at shelters. This study aimed to describe times that lost cats were missing for, search methods associated with their recovery, locations where found and distances travelled. A retrospective case series was conducted where self-selected participants whose cat had gone missing provided data in an online questionnaire. Of the 1210 study cats, only 61% were found within one year, with 34% recovered alive by the owner within 7 days. Few cats were found alive after 90 days. There was evidence that physical searching increased the chance of finding the cat alive (*p* = 0.073), and 75% of cats were found within 500 m of the point of escape. Up to 75% of cats with outdoor access traveled 1609 m, further than the distance traveled by indoor-only cats (137 m; *p* ≤ 0.001). Cats considered to be highly curious were more likely to be found inside someone else’s house compared to other personality types. These findings suggest that thorough physical searching is a useful strategy, and should be conducted within the first week after cats go missing. They also support further investigation into whether shelter, neuter and return programs improve the chance of owners recovering missing cats and decrease numbers of cats euthanized in shelters.

## 1. Introduction

Thirty percent of American households have a pet cat [1], and 15% of cat owners lose their pet at least once in a 5-year period [2]. Many of these animals are not reunited with their owner, despite the owner desiring them back. A common outcome for a proportion of missing cats is to be taken into a shelter or municipal animal control facility. Many are ultimately euthanized if not reclaimed after a standard holding period that varies among shelters but is usually between 3 to 5 business days [3,4,5,6].

Of stray animals entering shelters in USA and Australia, reported reclaim percentages for cats are typically 2–4% compared to reclaim percentages for dogs which usually range from 26–40%, but can be as high as 90% [6,7,8]. Cats are 13 times more likely to return to owners by means other than a visit to a shelter [9]. For example, reunification may occur directly via the general public if the cat has identification such as an ID tag, or as a result of signage (e.g., lost and found posters). Alternatively, local neighborhood searches and owner-initiated trapping may be successful [2,10].

However, there is little published information to inform searches for lost cats, particularly regarding the likely location of the pet and effective search methods. Current literature informs on different methods used to find lost pets [10], but the authors could find no peer-reviewed studies reporting the efficacy of methods used for finding missing cats, the most common locations missing cats were found, and the distance that lost cats travelled from where they went missing. This information is important so searches for lost pets are more effective at reuniting cats with their owners, therefore reducing euthanasia of unclaimed cats in shelters and municipal facilities.

The aim of the current study is to address gaps in the literature by reporting search methods used to find missing cats, methods most often associated with locating missing cats, and the location and distance travelled by missing cats that were found. It is anticipated that this information would assist in improving recovery percentages for lost cats.

## 2. Materials and Methods 

This retrospective case series of owned cats that had become missing was conducted with cases (episodes of cats becoming lost, hereafter referred to as ‘cats’ or ‘missing cats’) recruited using a convenience sample, including snowball sampling. The study was approved by The University of Queensland’s (UQ) Human Ethics Committee (approval number 2016000737). The questionnaire format and content was formulated by a team consisting of a 5th year research elective student and staff within The University of Queensland’s School of Veterinary Science and members of Missing Pet Partnership (MPP), a not-for-profit organization in the United States involved in reuniting lost companion animals with their owners.

This study was conducted from June through to August 2016 via an online questionnaire, using commercial survey software (SurveyMonkey^®^, San Mateo, CA, USA), which was promoted by MPP. Promotional methods used were primarily through social media, word-of-mouth, and on the organization’s own website. Potential respondents were told that the questionnaire was to be completed by people over 18 years of age. Each completed questionnaire collected data for one cat that went missing once (i.e., for one case). If the respondent had multiple cats that went missing, they could complete the questionnaire separately for each cat. If the same cat had gone missing multiple times, the respondent could complete the questionnaire separately for each episode of their cat going missing.

Question formats used a combination of multi-choice answers, free-text answers as well as rating scales for questions that were more qualitative/emotive (e.g., describing the cat’s personality type). The questionnaire consisted of 47 questions relating to the following:

Signalment and basic history of lost cat—age, neuter status, duration of pet ownership, where it was obtained from, possession of any type of identification, experience with the environment outside, where it lived, personality type, degree of human-pet bond.

Circumstances in which it went missing—age at time it went missing, number of people home at time it went missing, how long it was missing for, approximate time when it went missing, where did it go missing from, number of hiding places around where it went missing;

Search methods used to locate the cat—how long was the search for, if cat was still being searched for, search methods used to locate the cat split into five broad categories of ‘physical search’, ‘placing an advert’, ‘contacting professional help’, ‘trap techniques’, ‘recovered via identification device’, ‘waiting’, in which each had sub-headings with more specific options and search methods that the participant thought was most effective;

Circumstances in which the cat was found (if applicable)—whether the cat was found or not, condition and behavior of the cat when found, where the cat was found, how far the cat was found from where it went missing.

Respondents were also asked to rank their cat for each of four cat personality traits consisting of curious/clown cat, aloofness, cautiousness and timidity/fearfulness [11] on scale from 1 to 5 where 1 indicated ‘not at all’ and 5 indicated ‘perfectly describes my cat’s personality’.

The questionnaire was pilot tested by several UQ and MPP colleagues for clarity one month before launching, and it was subsequently modified. Responses were accepted from anywhere in the world.

Statistical analyses were completed using Stata (version 14, StataCorp, College Station, TX, USA). Each cat’s possible outcomes were: found alive, found dead, and not found by the time the respondent completed the questionnaire. Outcomes were allocated to cats based on an algorithm that used responses to four study questions (Table 1). Times from going missing to being found were as provided by respondents; for cats not found, time intervals were right-censored at the time the respondent completed the questionnaire using their reported time since the cat went missing. Cats whose times were not provided were excluded from analyses of time to being found alive (i.e., cats found, but time missing was not provided; and cats not found, and time since cat went missing was not provided). Cats whose times ended on the same day as they went missing were allocated a time of 0.5 day. Cumulative incidences of being found alive by time from when the cat went missing were then calculated using competing risks survival analyses, where being found dead was the competing event, using Stata’s-stcompet-command. These cumulative incidences described the probability that an individual cat would be found alive by time since the cat went missing. Cumulative incidence values are always on or between 0 and 1 (100%). Ninety-five percent point-wise confidence intervals were calculated using formula 4 described by Choudhury [12]. Potential determinants of being found alive were assessed using competing risks regression, using Stata’s-stcrreg-command. These used the method of Fine and Gray [13] and modeled the sub-hazard of being found alive, calculated such that cats that experienced the competing risk (i.e., were found dead) then were no longer at risk of being found alive. If the sub-hazard ratio is above 1 for cats exposed to a factor relative to non-exposed cats, the sub-hazard and the cumulative incidence are higher in exposed cats.

Distances from where the cat went missing to where it was found were compared between cats found alive and those found dead using the Mann-Whitney (or Wilcoxon) rank sum test, using Stata’s-ranksum-command. Distances were also compared by cat’s prior exposure to outdoors using the Kruskal-Wallis test using Stata’s-kwallis-command and pair-wise comparison between those never allowed outside and each other category were performed using Wilcoxon’s rank sum tests. Respondents’ ranks for each of the four cat personality traits were compared by location where the cat was found using Kruskal-Wallis tests. Responses recorded as 0 were reallocated as 1 (‘not at all’). Responses of 5 indicated ‘perfectly describes my cat’s personality’; responses recorded as numbers greater than 5 were excluded from analysis of that trait. For Kruskal-Wallis tests, chi-squared statistic adjusted for ties were used.

Distributions of study cats were expressed as percentages of cats whose status for the attribute was recorded or could be deduced from the provided data. For questions where the respondent could choose one or more options, cats were only included when calculating percentages if at least one option was selected.

## 3. Results

### 3.1. Demographics of Cats

In total, 1210 cats were recruited from 10 June to 17 August 2016. Of the 686 cats where country of residence was specified, most of the cats resided in the USA (59%), Australia (20%), and Canada (14%). Cats from 12 other countries were also recruited; these were from the United Kingdom (30 cats), and between 1 and 4 cats from each of Belgium, Bermuda, Brunei, Finland, France, Mexico, Philippines, Scotland, Spain, and South Africa.

Most cats were male (57%), most had been neutered (96%), and had been acquired from a shelter or rescue (33%) or from family or acquaintance (18%) (Table 2). Half had gone missing 2 years or less prior to the date when the respondent completed the questionnaire (5th and 95th percentiles 4 days and 14 years, respectively).

The majority of cats (67%) were 1 to 7 years old when they went missing (Table 2). Just over half of the cats (57% or 681/1196) had at least one form of identification (collars with no ID tag were not regarded as identification) and microchip was the most common form used (46% of all cats) (Table 2). Other forms of identification used were, in decreasing order of frequency, collars with an ID tag, identification tattoo, and collar with a tracking device (Table 2).

Nearly 90% of respondents strongly agreed with statements that they were attached to their missing cat and regarded it as a family member (Table 3). The cat’s dwelling was most commonly a house, townhouse or condominium with a yard (77%) (Table 2).

In the 6 months period prior to going missing, most cats were indoor-outdoor cats (69%), with 46% of all cats being allowed outdoors unsupervised. Some (28%) of respondents reported their cat was kept strictly indoors and never allowed outdoors (Table 2). Of these cats, 42% had experience more than 6 months previously of the outdoors (Table 2) and of all study cats, 85% had previous and/or recent experience of the outdoors.

Respondents ranked on a scale from 1 to 5 how well each of four personality descriptions fitted their cat, where 1 indicated ‘not at all’ and 5 indicated ‘perfectly describes my cat’s personality’. Ranks varied widely for ‘curious/clown cat’ and ‘cautious cat’ (*n* = 1049 and 1022 valid responses, respectively) but 62% of cats were scored 1 or 2 for ‘aloof cat’ (indicating that they were not aloof), and 73% of cats were scored 1 or 2 for ‘timid/fearful cat’ (indicating that they were not timid or fearful; *n* = 1004 and 1011 valid responses, respectively).

### 3.2. Circumstances Preceding and Leading to Cats Going Missing

At the time the cat went missing, 95% were in a familiar location and similar proportions of cats were indoors (37%), outdoors (25%), or had indoor-outdoor access (31%) (Table 4). Of those cats that were indoors at time of disappearance whose escape route was known (*n* = 403), the majority escaped through an open door or garage (74%). Of cats that were outdoors whose escape route was known (*n* = 169), most escaped while outdoors without supervision (64%) (Table 4). Of those cats that escaped while being transported (*n* = 25), the majority escaped from the carrier (52%) (Table 4). Most cats that disappeared from unfamiliar locations and whose escape route was known (*n* = 57) escaped shortly after moving into a new house (51%) or from a friend or pet-sitter’s home (34%) (Table 4).

### 3.3. Probabilities of Being Found Alive by Time Since Lost

**Figure 1:**
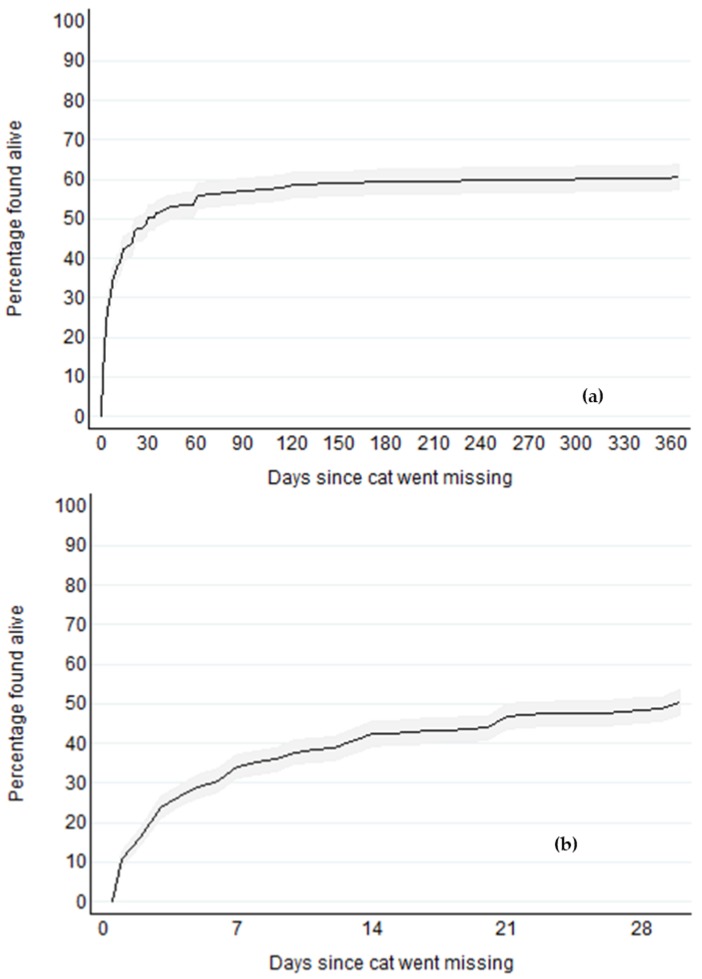
Cumulative percentages of missing cats found alive (cumulative incidences) by days since cat went missing; shaded band indicates 95% point-wise confidence interval. Being found dead was treated as a competing risk. (**a**) first 365 days; (**b**) first 30 days.

Of the 1210 cats, the outcome could be determined for 1075 (Table 1), and for 1044 of these cats, time from going missing to being found or, for cats not found, time to right-censoring (i.e., time since the cat went missing), were provided. Probabilities of an individual cat being found alive were calculated using these 1044 cats. Probabilities of an individual cat being found alive (i.e., cumulative incidences) by time since the cat went missing are shown in Figure 1a,b. Of missing cats, approximately one-third (34%; 95% CI 31% to 37%) were found alive by day 7, 50% (95% CI 47% to 53%) by day 30, and 56% (95% CI 53% to 59%) by day 61. There was little increase in probability of being found alive after day 61. By one year, 61% of missing cats had been found alive (95% CI 57% to 64%) and this had increased to only 64% (95% CI 60% to 67%) by 4 years.

Of these 1044 cats, 601 were found alive, and the median time they remained lost was 6 days (25th and 75th percentiles 2 and 21 days, respectively). Of the 17 cats that were found dead, the median time they remained lost was 21 days (25th and 75th percentiles 6 and 91 days, respectively). For the 426 cats not found at the time the questionnaire was completed, the median time they had been lost for was 365 days (25th and 75th percentiles 35 and 1096 days, respectively).

### 3.4. Search Methods Used, Perceived Successful Methods, and Associations with the Cat Being Found Alive

To assess what methods were used to find each cat, respondents were offered five possible search methods—physically did a search, advertised, contacted a facility or sought professional help, used a trapping technique, and identification device. Respondents could select one or more of these, or they could indicate that they just waited for the cat to return. Of the 1044 cats whose time from going missing was provided, for 991, the respondent indicated what was done to find the cat. Distributions of these cats by search method and cat’s outcome are shown in Table 5. Of respondents that used each method, the percentage who nominated that method as one that helped the most are also shown. For these 991 cats, the respondent used 0 (1%), 1 (18%), 2 (21%), 3 (35%), 4 (20%) or 5 (5%) of the five possible search methods. A physical search was the most common search method used (used for 96% of cats) and many people also advertised (74%) (Table 5). Approximately half of respondents (57%) contacted a facility or sought professional help, with most of these contacting (83%) and/or visiting (53%) a shelter (Table 5).

Outcomes are reported by search method in Table 5. Where a physical search was conducted, 59% of cats were found alive. There was some evidence that cats were more likely to be found alive if physical searching was conducted than if no physical searching was conducted (*p* = 0.071; Table 5). Percentages of cats that were found alive were similar (57% to 62%) for all of the particular types of physical searching other than ‘drove around the area’ (52%; Table 5). The relative time taken to find the cat alive due to the particular search method, relative to the time taken to find the cat alive by other methods or waiting, is shown in the right-hand column in Table 5 (‘Where this method was used and the cat was found alive, % of those cats where this method helped the most’). A high percentage indicates that the particular search method was generally more rapid in resulting in the cat being found alive relative to the time that the alternative other methods and types and /or just waiting collectively took to result in the cat being found alive. Relative to other methods or waiting, the most rapid relative times taken to find the cat alive using a physical search strategy were when the search methods included ‘spoke with neighbors and asked them to look or assist in the search for my cat’, ‘walked around the area at night, using a flashlight (or spotlight)’, ‘asked and received neighbors’ permission to search their property using a slow methodical search’ and ‘searched my yard or the immediate area’ (Table 5).

Where any type of advertising were used or if the owner contacted a facility or sought professional help at any stage, the cat was less likely to be ultimately found alive compared to if that search method was not conducted (*p* < 0.001 for both; Table 5). For most of the particular types of these methods, between 41% and 55% of cats were found alive (Table 5). Of the advertising strategies, highest percentages of cats were found alive (52% to 55%) when fliers were distributed or posters mounted.

For cats with an identification device (microchip, ID tag etc.) where the owner contacted a facility or sought professional help, there was no evidence that cats were more likely to be found alive (*p* = 0.331). Similarly, there was also no evidence that cats were more likely to be found alive with use of trapping techniques (*p* = 0.307). For the particular trapping techniques and identification devices, 50% to 65% of cats were found alive (Table 5). These associations between the various search methods and being found alive are univariable and did not account for time since the cat went missing when the search method was implemented.

### 3.5. Distances to Where Cat Found

**Figure 2:**
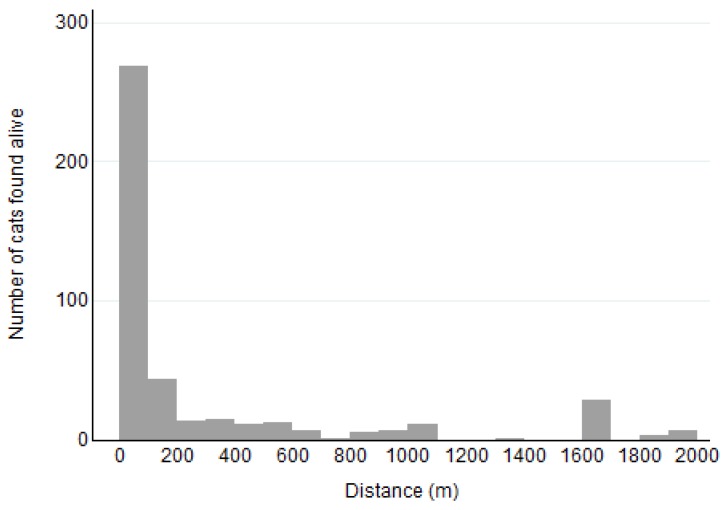
Distances at which missing cats were found from where they went missing for cats found alive (*n* = 434); a further 43 cats were found between 3000 and 25,000 m from where they went missing.

**Figure 3:**
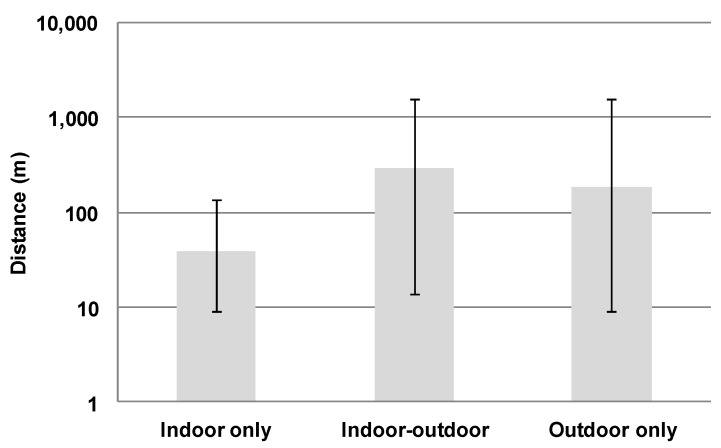
Median distances at which missing cats were found from where they went missing by outdoor experience in the 6 months prior to the cat going missing (indoor only *n* = 164; indoor-outdoor *n* = 150; outdoor only *n* = 15); error bars indicate 25th and 75th percentiles.

Of the 602 cats found alive, the distance from where the cat went missing to where it was found was recorded for 479 cats. Of these cats, 2 had implausibly high distances for cats to travel unassisted (>321 km) and these were disregarded. For the remaining 477 cats found alive, the median distance was 50 m (25th and 75th percentiles 9 and 500 m, respectively) (Figure 2) with the maximum distance being 25 km. For the 17 cats found dead where distance was recorded, the median distance was 200 m (25th and 75th percentiles 91 and 500 m, respectively), with the maximum distance being 5 km (*p* = 0.07). Of cats found alive, distances varied by outside experience in the 6 months before going missing (overall *p* < 0.001). Those cats that were indoor-outdoor and allowed outside unsupervised had longer distances compared with indoor cats that were never allowed outside (*p* ≤ 0.001) (Figure 3). The median distance for indoor-outdoor cats was 300 m (25th and 75th percentiles 14 and 1609 m (i.e., 1 mile), respectively; *n* = 150 cats) and indoor-only cats was 39 m (25th and 75th percentiles 9 and 137 m, respectively; *n* = 164 cats). Median distance for outdoor-only cats was 183 m (25th and 75th percentiles 9 and 1609m; *n* = 15 cats). These did not differ significantly from indoor-only cats (*p* = 0.173).

### 3.6. Locations Where Cats were Found and Cats’ Demeanors When Found

Of the cats that were found alive, the vast majority were found outside (83%) (Table 6). This was followed by the option offered as ‘cat being found inside someone else’s house’ (11%), inside the house where they lived (4%), and inside a public building (2%) (Table 6), therefore less than 2% of found cats were in a shelter or municipal animal facility. Specific locations are also shown in Table 6. Under ‘other’, respondents commonly described their cat as “coming home by itself” or being found waiting at the front door or entrance points to the house. Other common responses were finding their cat near their house, their neighbor’s house, or in nearby woods, vegetation or roads.

**Figure 4:**
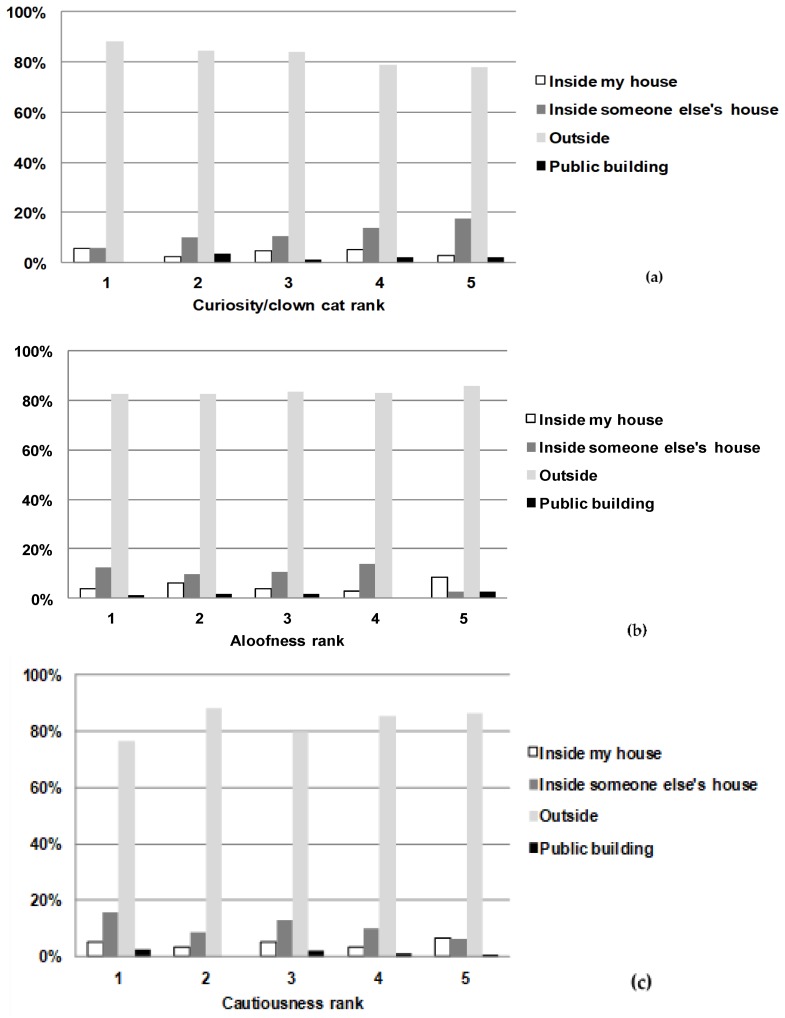

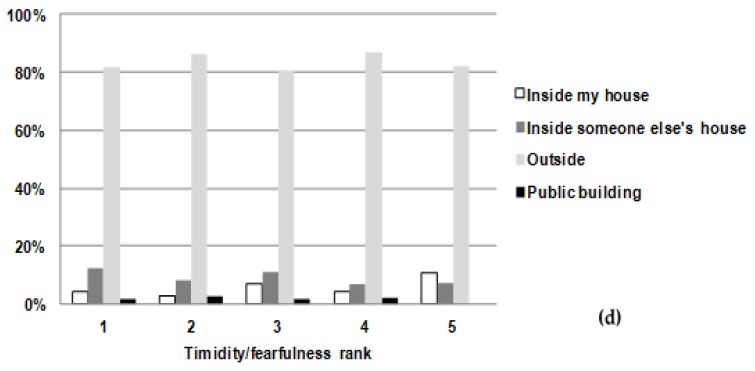
Locations where missing cats were found by (**a**) Cat’s curiosity/clown cat rank (*n* = 561); (**b**) Cat’s aloofness rank (*n* = 541); (**c**) Cat’s cautiousness rank (*n* = 546); (**d**) Cat’s timidity/fearfulness rank (*n* = 548). Respondents ranked their cat on a scale from 1 to 5, where 1 indicated the cat did not have the personality trait (‘not at all’) and 5 indicated ‘perfectly describes my cat’s personality’.

When looking at cats’ personalities with respect to location where the cat was found, for curious/clown cat, 6% of score 1 cats (indicating that they were not curious) were found inside someone else’s house compared with 10% of score 2 cats, 10% of score 3 cats, 14% of score 4 cats, and 17% of cats that were considered very curious (score 5) (*p* = 0.005) (Figure 4a). For the personality traits aloofness (*p* = 0.848), cautiousness (*p* = 0.123) and timidity/fearfulness (*p* = 0.192), there were no consistent associations between percentages of cats found in each location and the personality trait (Figure 4b–d).

Of the 585 cats that were found and at least one behavior when found was reported, only a minority (10%) of cats hissed or acted in a manner of a feral cat. Most cats appeared scared (50%), quiet but alert (30%), and/or friendly/relaxed (25%). A quarter of cats were vocalizing or meowing when found, and 9% were sick or injured when found.

## 4. Discussion

The outcomes of the present study provide valuable information on successful search methods to recover missing cats, likely locations where they are found, distances travelled and ways in which cats go missing. It also provides information on the likely locations where missing cats could be found based on their personalities and outdoor experience.

### 4.1. Locations of Missing Cats and Distances Travelled

An important finding was that most cats were found near their point of escape, with half being found within 50 m and three quarters within 500 m. This is in line with recent studies that have found most cats (66%) were recovered by either returning home on their own or being found in the neighboring vicinity (7%) [10]. In the current study, indoor-only cats tended to be found nearer to their escape point compared with indoor-outdoor and outdoor-only cats, highlighting the importance of tailoring the distance radius of a physical search to the outdoor experience of the cat. A larger radius of search may be required to find cats that are indoor-outdoor or outdoor-only under no supervision (distance traveled 1600 m for up to 75% of cats) compared with strictly indoor cats (distance traveled 137 m for up to 75% of cats) and thus may require investing more time, effort and resources. Therefore, a search in a 200-m radius is more likely to suffice in a search for a missing indoor-only cat, but a search for an outdoor or indoor-outdoor cat is more likely to have to be extended up to 2 km radius or more.

Cats most commonly escaped into areas that had 7 or more places to hide in, such as nearby shrubbery, under motor vehicles, decks, porches, or in sheds. Most cats when found outside were hiding under porches, cars or under objects near the house where they resided. Of these cats, 18% were found directly outside the entrance points of the owner’s house (e.g., by the door or by the porch) or described as “simply came home”, which is in line with American Society for the Prevention of Cruelty to Animals (ASPCA) observations that while “dogs are sought, cats come home” [2,8]. Our study has also shown that the personality of the cat may be indicative of the likely location where it will be, with cats described as highly curious more likely to be found inside someone else’s house or in public buildings. The percentage of missing cats found outside decreased steadily with increasing curiosity scores, and the percentage that were found inside someone else’s house increased steadily with increasing curiosity scores.

The finding that owned cats are often found not far from where they go missing provides evidence to support shelter-neuter and return (SNR) strategies, also known as return to field and cat diversion. This is a process whereby stray cats that are likely to be euthanized in shelters or animal control facilities are temporarily sheltered, then neutered and returned to the original location they were found as an alternative to euthanasia [14]. The rationale for this is that lost owned, semi-owned and community cats who are cared for by people [4,15,16], do not travel far from the location where they are fed. Our results also support returning unidentified owned, semi-owned and community cats to their home territory as an alternative to euthanasia. Shelter-neuter and return strategies markedly reduce euthanasia in facilities with large intakes of stray cats, and are also reported to reduce intake of stray cats [14].

### 4.2. Search Timing and Methods

Of the cats found alive, almost all were recovered within the first 2 months, with approximately half recovered within the first 7 days. This was probably largely because cats that were in situations making them easier to find were, accordingly, found more quickly. However, it may also have been partly because finding cats soon after they go missing prevents such cats from being harmed or subsequently becoming irretrievably lost if not found quickly. Physical searching, advertising, and contacting a facility or seeking professional assistance were the 3 most common search methods used. Physical searching was used most commonly, and there was some evidence that the probability of recovering a missing cat alive is increased if this method is conducted. More specifically, the most successful physical search strategies were ‘searched my yard and the immediate area’, ‘while I was looking for my cats, I called its name’, ‘walked around the area during daylight hours’ and ‘spoke with neighbors and asked them to look or assist in the search for my cat’.

Cats were less likely to be found alive if any type of advertising were used, or if the owner contacted a facility or sought professional help. As these strategies are unlikely to reduce the probability of missing cats being found alive, these observed associations were probably due to selective implementation of these strategies more commonly when lost cats had not been found for some time, and thus already had reduced chances of being found alive. As our study was retrospective, it was not possible to collect the chronology of search strategies. These data may be collectable in prospective studies. If available, confounding for this reason should be reduced if effects of various search strategies were assessed using survival analysis with search strategies fitted as time-varying covariates. In addition, for many lost cats, multiple search strategies are used, and multivariable models would be useful when assessing the effect of particular search strategies

Fifty seven percent of cats were recovered alive if pet detective or volunteer lost pet recovery service was used. This probably reflects the ability of these services to provide appropriate advice and use efficient methods for the recovery of the missing cat, such as conducting a thorough physical search, distributing flyers and using a humane trap. Although less than 2% of cats found alive were in a shelter, 36% of respondents who found their cat alive and visited or called a shelter rated it as one of the two most helpful methods, possibly because shelter staff provided advice on appropriate search methods to be used.

Only approximately half of the study cats were microchipped, emphasizing the importance of minimum holding periods in shelters and animal control facilities to allow time for owners of non-microchipped cats to search for them. In the current study, microchipping was not found to improve the chance of missing cats being found alive. This may have been because few lost cats were taken to a facility where microchips can be read (shelter, animal control facility or veterinary clinic). It may also have been due, in part, to the fact that for recovery via microchipping, it must have updated owner’s contact details. For a substantial proportion of microchipped dogs and cats (37% in one study [17]), the chip was registered to incorrect owner data such as the cat’s previous owner, or incorrect or disconnected phone lines, or the microchip was not properly registered. The proportion of animals reclaimed from a shelter declines significantly if microchipped animals have data problems (41% compared to 75% for microchipped cats with no such data problems [17].

### 4.3. Respondent and Missing Cat-Related Demographics

The respondents in this study seemed to have a strong human-pet bond with their cat compared with the general population. Of all respondents, 90% answered they were strongly attached to their pet and regarded them as a family member. This is a higher proportion than in other studies of pet owners in Western countries describing a strong human-pet bond [18,19,20,21], and only 35–45% of respondents in one survey would have regarded their pet (dog or cat) as a family member [18]

### 4.4. Limitations

There were likely to have been recall errors in the reported study data as a substantial proportion of respondents (71%) had lost their cat more than 6 months ago prior to the date when they completed the questionnaire, with the median duration being 731 days (i.e., 2 years). This may have caused misclassification of related variables, possibly distorting measures of association and reducing the statistical power of the study. The strong human-animal attachment respondents reported having with their cat could also mean that respondents and thus their responses are not representative of the general population, but of a sub-sample of the general population that is more closely bonded to their cat than average. The generalizability of the findings may thus be limited, e.g., the study population may have comprised people more likely to put in more effort to find their cat than the average pet owner.

The exact proportion of cats found in a shelter or animal control facility was not able to be ascertained because of an oversight in the questionnaire design, however based on results, this represented less than 2% of cats found alive. Analysis of recovery by microchipping was challenging to interpret because any benefits of microchipping would only be expected if the cat was taken to a location where a scanner was available. Benefits of microchipping could also be affected by the duration of the minimum holding period for cats in the facility, and accuracy of the owner contact details in animal identification databases.

Our analyses did not sub-divide cats into those that were indoor-only cats versus those that had had outdoor experience. Cat’s experience with the outside world could affect their behaviors when displaced with respect to distance travelled, location found and likelihood of recovery. Future studies in this field could look into analyzing how these two groups of cats behave and how that may affect the effectiveness of search methods and probabilities of recovery.

In this study, cats found dead were found further away than cats found alive. It is possible this could be a misrepresentation if, for example, the cats found deceased were actually admitted into a shelter, animal control facility or veterinary clinic by a member of the public before the owner was notified. Similarly, those found deceased close to home were less likely to be classified as “missing” by their owners. As the sample group was small (1.7% of total cats in the study), analysis for this category of cats was limited.

## 5. Conclusions

The most significant findings of this study were that a thorough physical search is likely to increase the chances of finding cats alive and most cats are found within a 500 m radius of their point of escape. Cats that were indoor-outdoor and allowed outside unsupervised traveled longer distances compared with indoor cats that were never allowed outside. These findings support previous studies as well as unpublished empirical reports that cats do not travel far from their homes, and are best found with thorough physical searching, the radius varying depending on the missing cat’s previous outdoor experience. There is about a 33% chance that a missing cat will be found within 7 days, and about 56% by 2 months; few cats are recovered after that. In addition, cats with a predominantly curious personality type may have a predilection to hide or be found in neighboring houses. These findings may help inform searchers and ultimately increase the chances of reuniting lost pets with their owners. In addition, the information in this study, especially the fact that lost cats usually do not travel far from where they were lost, may provide justification for SNR programs. Future research in the field is required towards improving return rates for missing cats and to provide further evidence regarding the benefit and applicability of SNR programs in reducing cat euthanasia in shelters and animal control facilities.

## Figures and Tables

**Table 1:** Algorithm for allocating outcome categories to study cats.

When Your Cat Was Found, Where Was Your Cat Found?	Please Indicate Whether or Not Your Cat Was Found.	At This Moment, Which Option Best Describes the Searching Situation Regarding Your Cat?	If You Found Your Missing Cat, What Are the Two Primary Methods or Resources that Helped You the Most?	*n* ^1^	Allocated Outcome
Location selected	My cat was found	My cat has been found alive		583	Found alive
No location selected	My cat was found	My cat has been found alive		4	Found alive
No location selected		My cat has been found alive		15	Found alive
Location selected	My cat was found	Sadly, my cat was found dead		17	Found dead
No location selected	My cat has not been found	Sadly, my cat was found dead		3	Found dead
No location selected		Sadly, my cat was found dead		1	Found dead
No location selected		I am still searching continuously		11	Not found
No location selected	My cat has not been found	I am still searching continuously	I did not find my cat	69	Not found
No location selected	My cat has not been found	I am still searching continuously		39	Not found
No location selected	My cat has not been found	I am still searching on and off	I did not find my cat	68	Not found
No location selected	My cat has not been found	I am still searching on and off		48	Not found
No location selected	My cat has not been found	I have stopped searching and did not find my cat	I did not find my cat	111	Not found
No location selected	My cat has not been found	I have stopped searching and did not find my cat		70	Not found
No location selected	My cat has not been found	Sadly, my cat was found dead	I did not find my cat	5	Not found
No location selected		I am still searching on and off		13	Not found
No location selected		I have stopped searching and did not find my cat	I did not find my cat	1	Not found
No location selected		I have stopped searching and did not find my cat		17	Not found
Location selected		I am still searching continuously		1	Discordant ^2^
Location selected	My cat was found			1	Discordant ^2^
Location selected	My cat was found	I am still searching continuously		4	Discordant ^2^
Location selected	My cat was found	I am still searching on and off		2	Discordant ^2^
Location selected	My cat was found	I have stopped searching and did not find my cat		1	Discordant ^2^
Location selected	My cat was found	My cat has been found alive	I did not find my cat	1	Discordant ^2^
Location selected	My cat was found	My cat has been found alive	I did not find my cat	1	Discordant ^2^
Location selected	My cat was found	Sadly, my cat was found dead	I did not find my cat	3	Discordant ^2^
Location selected				1	Discordant ^2^
Location selected		I have stopped searching and did not find my cat		1	Discordant ^2^
No location selected	My cat was found	I have stopped searching and did not find my cat	I did not find my cat	1	Discordant ^2^
No location selected				118	Unclassifiable
Total				1210	

^1^ Number of cats allocated to each outcome category; ^2^ Responses were discordant so no outcome was allocated.

**Table 2:** Distributions of demographic values for 1210 cats that went missing.

Variable	% (*n*) ^1^
**Cat’s sex**	
Male	57% (690)
Female	43% (513)
Not recorded	7
**Neuter status**	
Neutered	96% (1134)
Intact	4% (53)
Not recorded	23
**Cats’ age when it went missing**	
Kitten (0–11 months)	10% (115)
Adult (1–7 years)	67% (796)
Senior (8 years or older)	24% (282)
Not recorded	17
**Where was the missing cat acquired from**	
Animal shelter or rescue	33% (402)
From a veterinarian	2% (30)
Breeder	5% (62)
Pet shop	4% (52)
Advertisement in newspaper	1% (17)
Online or from the Internet	3% (38)
Family member or friend or acquaintance	18% (219)
As a gift	1% (9)
Found as a stray in a public location	10% (121)
Appeared as a stray at my home	10% (118)
Other	11% (136)
Not recorded	6
**Type of identification present when cat went missing ^2^**	
Microchip	46% (555)
Collar but no tag	12% 144)
Collar with ID tag	19% (229)
Collar with GPS-tracking device	0% (1)
Collar with radio-tracking device	0% (0)
Identification tattoo	5% (56)
No identification (i.e., none of the above)	37% (443)
No identification or only collar but no tag	43% (515)
Multiple inconsistent options (i.e., no identification and a form of identification) selected	4
Not recorded	10
**Missing cat’s experience with the outside world in the previous 6 months before going missing**	
Indoor—strictly never allowed outside	28% (322)
Primarily indoor—only allowed outside on a leash	5% (58)
Primarily indoor—only allowed outside in an enclosure porch or “catio”	6% (75)
Primarily indoor—allowed outside under supervision	12% (138)
Indoor-outdoor—allowed outdoors unsupervised	46% (538)
Outdoor—strictly never allowed inside	3% (35)
Not recorded	44
**For strictly indoor cats in the previous 6 months before going missing, prior experience in their life with being outdoors**	
No, strictly never allowed outside	37% (102)
Never allowed outside, but has escaped outside at least once	22% (61)
Previously allowed outdoors on a leash	3% (9)
Previously allowed outdoors in an enclosure, porch or “catio”	3% (7)
Previously allowed outdoors supervised	5% (14)
Previously allowed outdoors unsupervised	7% (19)
Previously an outdoor cat (e.g., adopted as a stray cat)	24% (67)
I don’t know (e.g., adopted as an adult cat and has an unknown history outdoors)	41
Not recorded	2
Not strictly indoor cat in the previous 6 months before going missing or outdoors experience in that period not recorded	888
**For cats that had been indoor-only or primarily indoor for the previous 6 months before going missing, prior experience in their life with being outdoors**	
No, strictly never allowed outside	23% (121)
Never allowed outside, but has escaped outside at least once	16% (81)
Previously allowed outdoors on a leash	7% (36)
Previously allowed outdoors in an enclosure, porch or “catio”	7% (34)
Previously allowed outdoors supervised	16% (81)
Previously allowed outdoors unsupervised	9% (46)
Previously an outdoor cat (e.g., adopted as a stray cat)	23% (117)
I don’t know (e.g., adopted as an adult cat and has an unknown history outdoors)	62
Not recorded	15
Not indoor-only or primarily indoor cat in the previous 6 months before going missing or outdoors experience in that period not recorded	617
**Dwelling location when cat went missing**	
Residential—mainly apartments	11% (123)
Residential—mainly houses	73% (794)
Commercial	1% (9)
Acreage/hobby farm	4% (44)
Rural/farmland/ranches	5% (59)
Wilderness/forest	2% (20)
Mixed residential and forest/wilderness	1% (15)
Mixed residential and another type of dwelling	1% (7)
Desert	0% (3)
Other ^3^	1% (10)
Not recorded	113
**Dwelling type when cat went missing**	
House/townhouse/condominium with a yard/garden	77% (831)
House/townhouse/condominium without a yard/garden	3% (30)
Apartment—on the ground (1st) floor only	5% (57)
Apartment—on the 2nd–5th floor	6% (61)
Apartment—on the 6th–10th floor	0% (2)
Apartment—on 11th floor or above	0% (1)
Trailer/caravan/mobile home/camper	2% (18)
Farm/acreage	7% (70)
Other ^4^	0% (4)
Not recorded or invalid response	136

^1^ Within each demographic variable, numbers are expressed as percentages of valid responses for relevant cats; ^2^ Respondents could select more than one option. Percentages of the 1196 cats where at least one option was selected but multiple inconsistent options (i.e., no identification and a form of identification) were not selected; ^3^ Other dwelling locations were construction site (*n* = 1), mobile homes such as “trailers” (4),“bad neighborhood”(1), “cat friendly”(1), “residential but big gully”(1), “residential on the edge of farmland” (1), “university campus area—mainly houses/residential” (1); ^4^ Other dwelling types were “warehouse”(1), “hotel”(1) and “rural”(2).

**Table 3:** Distribution of owners’ relationships with their cats for 1210 cats that went missing (% (number of cats)). Numbers are expressed as percentages of those that responded.

Owners Relationship with Their Cat	Strongly Agree	Agree	Neutral	Disagree	Strongly Disagree	Not Recorded
I am very attached to my cat	88% (966)	10% (111)	2% (18)	0% (0)	0% (0)	115
I regard my cat as a family member	89% (978)	9% (101)	1% (13)	0% (0)	0% (3)	115

**Table 4:** Circumstances surrounding the disappearance of 1210 cats that went missing.

Question or Location when Went Missing (Total Number of Eligible Cats)	% (*n*) ^1^
**From where did your cat go missing? (*n* = 1210)**	
It was indoors	37% (403)
It was outdoors	25% (272)
It was an indoor-outdoor cat and disappeared	31% (338)
Escaped while being transported	2% (25)
It was at an unfamiliar location	5% (57)
Not recorded	115
**The cat was indoors when it went missing (*n* = 403)**	
Jumped from balcony	5% (19)
Jumped from a window	11% (42)
Escaped through an open door or garage	74% (272)
Escaped while outdoors without supervision	0% (1)
Accidental or unintentional transport off premises	1% (2)
Cat disappeared but never left indoor premises	1% (4)
Escaped through damaged window/door screen	6% (21)
Escaped through damaged cat enclosure	1% (6)
Other ^2^	1% (3)
Not recorded	33
**The cat was outdoors when it went missing (*n* = 272)**	
Escaped from enclosure outside in a yard or porch or “catio”	9% (15)
Escaped while on leash	4% (7)
Escaped while supervised outdoors	17% (28)
Escaped while outdoors without supervision	64% (109)
Accidental or unintentional transport off premises	6% (10)
Not recorded	103
**The cat escaped while being transported (*n* = 25)**	
Jumped out of vehicle through open door or window	16% (4)
Escaped when involved in a vehicular accident	4% (1)
Escaped from cat carrier	52% (13)
Escaped while on a leash	8% (2)
Escaped from owner’s arms	12% (3)
Other ^3^	8% (2)
**It was at an unfamiliar location (*n* = 57)**	
At boarding kennel or cattery	0% (0)
At a friend’s or pet sitter’s home	34% (19)
At a veterinary clinic	0% (0)
At the groomer	0% (0)
At a holiday location	11% (6)
Moved to a new home	51% (29)
Other ^4^	4% (2)
Not recorded	1
**At the time your cat went missing, which of the following best describes your situation? (*n* = 1210)**	
No one was home for up to 12 h	16% (179)
No one was home for 13–24 h	1% (16)
No one was home for more than 24 h	1% (13)
There was someone home	77% (869)
Away but there had been a house sitter for x number of days	4% (50)
Not recorded	83
**How many potential hiding places would you say there were in a 150 yard or 130 m radius (roughly 3–4 house radius) where your cat went missing? (*n* = 1210)**	
None	1% (5)
1 to 3	4% (31)
4 to 6	7% (50)
7 or more	89% (667)
Not recorded	457

^1^ Within each question, numbers are expressed as percentages of those that responded; ^2^ Other locations were “chimney” (*n* = 1), “neighbor’s door” (1), and “fell off roof” (1); ^3^ Other answers were “lost from vet office” (1) and “maybe the door was open a bit too long” (1); ^4^ Other answers were “cemetery” (1) and “my home back yard” (1).

**Table 5:** Distributions of cats by search method and cat’s outcome (found alive/found dead/not found). Results are reported for cats whose outcome could be determined and whose time from becoming lost to being found or, for cats not found, time since the cat went missing, was provided, and where the respondent indicated what was done to find the cat.

Search Method and Specific Type ^1^	% of Cats Where This Method Was Used (No./Denominator)	% of Cats by Outcome (N)			Sub-hazard Ratio for Cat Being Found Alive (95% CI) ^2^	*p*-Value	Where this Method Was Used and the Cat Was Found Alive, % of Those Cats Where This Method Helped the Most (No./Denominator) ^3^
		Found Alive	Found Dead	Not Found			
Physically did a search							
No	4% (38/991)	50% (19)	5% (2)	45% (17)	Reference category		
Any type of physical search	96% (953/991)	59% (566)	2% (15)	39% (372)	1.49 (0.97 to 2.29)	0.071	68% (326/482) ^4^
Specific types of physical search ^5^:							
Searched indoors	66% (628/952)	58% (364)	2% (12)	40% (252)			41% (131/316) ^6^
Searched my yard or the immediate area	95% (900/952)	59% (535)	2% (14)	39% (351)			50% (229/458)
Spoke with neighbors and asked them to look or assist in the search for my cat	85% (806/952)	57% (458)	2% (15)	41% (333)			57% (224/396)
Drove around the area	70% (671/952)	52% (348)	2% (11)	46% (312)			39% (117/300)
Walked around the area during daylight hours	92% (875/952)	58% (508)	2% (14)	40% (353)			49% (213/432)
Walked around the area at night, using a flashlight (or spotlight)	73% (694/952)	59% (408)	2% (13)	39% (273)			53% (190/356)
While I was looking for my cat, I called its name	93% (884/952)	58% (514)	2% (14)	40% (356)			34% (151/439) ^7^
When looking for my cat, I searched slowly and methodically	73% (692/952)	60% (415)	2% (12)	38% (265)			46% (169/365)
Asked and received neighbors’ permission to search their property using a slow methodical search	46% (438/952)	62% (273)	2% (9)	36% (156)			51% (126/245)
Advertised							
No	26% (255/991)	73% (185)	2% (4)	26% (66)	Reference category		
Any type of advertising	74% (736/991)	54% (400)	2% (13)	44% (323)	0.51 (0.42 to 0.61)	<0.001	52% (183/349)
Specific types of advertising ^5^:							
Distributed missing cat fliers	74% (545/734)	55% (301)	2% (12)	43% (232)			60% (159/265)
Posted fliers in local businesses	46% (341/734)	55% (186)	3% (9)	43% (146)			43% (71/166)
Used an automated phone call alert system such as LostMyKitty.com, Find Toto or Pet Amber Alert	13% (96/734)	46% (44)	1% (1)	53% (51)			44% (17/39)
Mailed lost pet postcards	5% (38/734)	29% (11)	3% (1)	68% (26)			38% (3/8)
Searched online for postings of found or adoptable cats	65% (480/734)	47% (227)	1% (7)	51% (246)			33% (65/199)
Used social media such as Facebook to spread the word that my cat was missing	68% (496/734)	49% (244)	2% (8)	49% (244)			46% (98/211)
Posted a lost pet advertisement in the online classifieds such as Craigslist, Kijiji or local newspaper online	35% (256/734)	41% (105)	2% (4)	57% (147)			36% (34/94)
Posted a lost pet advertisement in a newspaper	15% (107/734)	50% (53)	2% (2)	49% (52)			41% (21/51)
Posted on a missing pet database such as LostMyKitty.com, HelpingLostPets.com, or Tabby Tracker	36% (263/734)	43% (113)	1% (3)	56% (147)			33% (33/100)
Mounted missing cat posters (e.g., on poles, trees, etc.) which can be best described as: white 8 ½” × 11” (22 × 28 cm) paper posters	52% (381/734)	55% (209)	1% (5)	44% (167)			50% (95/190)
Mounted missing cat posters (e.g., on poles, trees, etc.) which can be best described as: neon color 8 ½” × 11” (22 × 28 cm) paper posters	17% (126/734)	54% (68)	2% (3)	44% (55)			44% (27/62)
Mounted missing cat posters (e.g., on poles, trees, etc.) which can be best described as: giant (22” × 24” or 56 × 60 cm) neon posters describing your missing pet in 5 words easily read in 5 seconds when driving past at 55 miles per hour or 90 kilometers per hour	15% (107/734)	52% (56)	4% (4)	44% (47)			63% (33/52)
Contacted a facility or sought professional help							
No	43% (430/991)	70% (301)	1% (6)	29% (123)	Reference category		
Any type of facility or professional help	57% (561/991)	51% (284)	2% (11)	47% (266)	0.53 (0.45 to 0.62)	<0.001	29% (69/242)
Specific types of facility or professional help ^5^							
Called shelters/rescue groups/municipal animal facility	83% (458/550)	50% (229)	2% (10)	48% (219)			35% (69/200)
Visited shelters/rescue groups/municipal animal facility	53% (291/550)	42% (122)	2% (6)	56% (163)			39% (44/114)
Contacted veterinarians	61% (338/550)	47% (160)	2% (7)	51% (171)			36% (49/136)
Contacted animal control or police department	50% (273/550)	47% (127)	2% (5)	52% (141)			30% (33/111)
Contacted Microchip company such as Home Again or 24PetWatch	29% (161/550)	43% (70)	2% (3)	55% (88)			23% (14/60)
Use an animal communicator or a pet psychic to tell me where my cat was located	11% (59/550)	39% (23)	3% (2)	58% (34)			45% (9/20)
Received assistance from a pet detective or volunteer lost pet recovery service/group	24% (130/550)	57% (74)	2% (3)	41% (53)			70% (49/70)
Used a trained search dog (cat detection or scent tracking)	0% (0/550)						^8^
Used a trapping technique							
No	80% (791/991)	58% (460)	2% (14)	40% (317)	Reference category		
Any type of trapping	20% (200/991)	63% (125)	2% (3)	36% (72)	0.91 (0.76 to 1.09)	0.307	65% (78/120)
Specific types of trapping ^5^:							
Used a “house as trap” method—open door/porch/garage/entry point that was monitored (baby monitor, driveway alarm, sat and watched the door) which allowed me to see when my cat came home.	30% (59/195)	54% (32)	3% (2)	42% (25)			34% (11/32)
Used a digital wildlife camera, video baby monitor or other surveillance camera to confirm my cat was hiding nearby	38% (75/195)	52% (39)	1% (1)	47% (35)			58% (22/38)
Used a humane trap or drop trap to capture my cat and bring it back home	88% (171/195)	65% (111)	1% (2)	34% (58)			72% (78/109)
Identification device ^9^ (for cats where the owner contacted a facility or sought professional help)							
No	38% (213/560)	48% (103)	2% (5)	49% (105)	Reference category		
Any type of identification device	62% (347/560)	52% (181)	2% (6)	46% (160)	1.12 (0.89 to 1.42)	0.331	18% (28/160)
Specific types of identification device ^5^:							
Microchip	61% (211/347)	46% (97)	2% (5)	52% (109)			47% (40/86)
Collar with ID tag	22% (75/347)	41% (31)	0% (0)	59% (44)			48% (12/25)
Collar with GPS-tracking device	0% (1/347)	0% (0)	0% (0)	100% (1)			^10^
Collar with radio-tracking device							^11^
It had an identification tattoo	4% (14/347)	57% (8)	0% (0)	43% (6)			67% (4/6)

^1^ Respondents could select more than one search method, and more than one specific type within a search method; ^2^ Univariable sub-hazard ratio for cat being found alive; being found dead was treated as a competing risk; time since the cat went missing when the search method was implemented was not accounted for; ^3^ Calculated only for those that selected the search method and nominated at least one method that helped the most; for identification device, only cats where the owner contacted a facility or sought professional help were included, and if the device was present at the time the cat went missing, the owner was assumed to have used that the search method; ^4^ For example, of the 566 cats where a physical search was conducted and the cat was found alive, for 482, the owner nominated at least one method (other than waited) as one of the two methods that helped the most. Of these 482, 68% nominated physical search. For the remaining 32% of cats, physical search was not nominated, so the cat was found alive but presumably not during a physical search; ^5^ Results for specific types are reported only for those that selected at least one specific type; ^6^ For example, of the 364 cats where a search indoors was conducted and the cat was found alive, for 316, the owner nominated physical search as one of the two methods that helped the most and provided additional information as requested. Of these 316, 41% nominated search indoors. For the remaining 59% of cats, search indoors was not nominated, so the cat was found alive but presumably not during a search indoors; ^7^ Of the 151 owners where this method (‘While I was looking for my cat, I called its name’) helped the most, 15% (22) selected ‘it came to me without meowing’, 38% (58) selected ‘it came to me and meowed’, and 54% (82) selected ‘it meowed back but didn’t come to me’. Respondents could select more than one of these options; ^8^ A trained search dog (cat detection or scent tracking) was not used for any cat; ^9^ Identification at the time the cat went missing; collar with no tag was not considered identification; ^1^^0^ The cat that had a collar with a GPS-tracking device was found dead; ^11^ No cat had a collar with a radio-tracking device.

**Table 6:** Circumstances in which missing cats were found for the 602 cats that were found alive.

Question (Total Number of Eligible Cats)	% (*n*) ^1^
**Where was your cat found? (*n* = 602)**	
My cat was found inside my house	4% (27)
My cat was found inside someone else’s house	11% (62)
My cat was found inside a public building	2% (9)
My cat was found outside	83% (485)
Not recorded	19
**If your cat was found inside your house, can you tell us more about where inside the house it was found? (*n* = 27)**	
Behind or under furniture	33% (3)
In basement	33% (3)
In furniture (e.g., box springs of couch)	11% (1)
Bedroom	22% (2)
Not recorded or invalid response	18
**If your cat was found inside your neighbor’s house, can you tell us more about where inside the house it was found? (*n* = 62)**	
Behind or under furniture	22% (7)
I don’t know	3% (1)
In basement	13% (4)
In ceiling	3% (1)
In furniture (e.g., box springs of couch)	6% (2)
On furniture (e.g., sofa)	3% (1)
Under floor or floorboards	9% (3)
Garage	28% (9)
Balcony	3% (1)
Barn	3% (1)
Kitchen	3% (1)
Bedroom	3% (1)
Not recorded or invalid response	30
**If your cat was found outside, can you tell us more about where it was found? (*n* = 485)**	
Found hiding under vegetation/shrubbery	16% (76)
In a garage	4% (17)
In a shed or barn	3% (13)
In a vehicle	0% (1)
In a wood-pile	0% (1)
In a yard	20% (95)
In farm or woods or forest	2% (8)
Storm drain or sewer	4% (19)
Under a garage	1% (6)
Under a shed	2% (8)
Under house	5% (25)
Under vehicle	3% (12)
In or between fencing	1% (5)
Under patio/deck/porch	10% (47)
In cat trap	4% (17)
On road-side	1% (6)
Up a tree	1% (4)
With a feral colony	1% (4)
Waiting outside home/house	19% (88)
Outside a building (e.g., apartment complex, commercial buildings)	3% (13)
Balcony	1% (3)
Not recorded	17

^1^ Within each question, numbers are expressed as percentages of valid responses.

## Abbreviations

The following abbreviations are used in this manuscript:

SNR	Shelter-neuter and return
UQ	The University of Queensland
ID	Identification
MPP	Missing Pet Partnership
